# Early Treatment with Fluvoxamine among Patients with COVID-19: A Cost-Consequence Model

**DOI:** 10.4269/ajtmh.22-0106

**Published:** 2022-11-15

**Authors:** Fergal P. Mills, Gilmar Reis, Lindsay A. Wilson, Kristian Thorlund, Jamie I. Forrest, Christina M. Guo, David R. Boulware, Edward J. Mills, for the TOGETHER Investigators

**Affiliations:** ^1^ Platform Life Sciences Inc., Vancouver, Canada;; ^2^ Research Division, Cardresearch–Cardiologia Assistencial e de Pesquisa, Belo Horizonte, Brazil;; ^3^ Department of Health Research Methods, Evidence, and Impact, McMaster University, Hamilton, Canada;; ^4^ Department of Medicine, University of Minnesota, Minneapolis, Minnesota

## Abstract

To date, two published randomized trials have indicated a clinical benefit of early treatment with fluvoxamine versus placebo for adults with symptomatic COVID-19. Using the results of the largest of these trials, the TOGETHER trial, we conducted a cost–consequence analysis to assess the health system benefits of preventing progression to severe COVID-19 in outpatient populations in the United States. A decision-analytic model in the form of a decision tree was constructed to evaluate two treatment strategies for high-risk patients with confirmed, symptomatic COVID-19 in the primary analysis: treatment with a 10-day course of fluvoxamine (100 mg twice daily) and current standard-of-care. A secondary analysis comparing a 5-day course of nirmatrelvir–ritonavir was also conducted. We used a time horizon of 28 days. Reported outcomes included cost-savings and hospitalization days avoided. The results of our analysis indicated that administration of fluvoxamine to symptomatic outpatients at high risk of progressing to severe COVID-19 was substantially cost-saving, in the amount of $232 per eligible patient and prevented an average of 0.15 hospital days per patient treated, compared with standard of care. Nirmatrelvir–ritonavir was also shown to be cost-saving despite its higher acquisition cost and provided savings to the healthcare system of $625 per patient treated. These findings suggest that fluvoxamine is likely to be a cost-effective addition to frontline COVID-19 mitigation strategies in many settings, particularly where access to nirmaltrevir–ritonavir or monoclonal antibodies is limited.

## INTRODUCTION

As efforts to scale up treatment of COVID-19 continue, repurposing existing medications that are widely available as generic formulations and that have well-understood safety profiles is particularly appealing.^1^ One such medication is fluvoxamine, a selective serotonin reuptake inhibitor (SSRI) and Sigma-1 receptor agonist^2^ with several potential mechanisms that may contribute to the treatment of COVID-19, including antiinflammatory and possible antiviral effects.^3^ To date, the results of two published randomized controlled trials (RCTs) have demonstrated that early administration of fluvoxamine is effective in reducing hospitalizations and/or progression to severe disease among outpatients with symptomatic COVID-19.^4^^,^^5^ These studies were conducted in the United States (*N* = 152) and in Brazil (*N* = 1,497). Both studies indicated improvements in time-to-recovery as well as reductions in emergency setting attendance and hospitalization. Real-world data also support the treatment benefits of fluvoxamine.^6^

In addition to the promise fluvoxamine holds for improving patient health outcomes, fluvoxamine may also help to reduce healthcare utilization and expenditures. Currently, as many as 20% of patients at high risk of COVID-19 disease progression require hospitalization, and 32% of these admissions require intensive care unit (ICU) admission.^7^ Of these ICU admissions, 20% require invasive mechanical ventilation.^8^ These developments are extremely costly; previous analyses report that an uncomplicated hospitalization costs US$10,557 in the United States, whereas a hospitalization with complications or a comorbidity costs US$14,887. Major complications increase estimated costs to US$21,943. Furthermore, hospitalizations requiring the use of a ventilator are longer and more expensive, with the cost per admission requiring ventilator support for > 96 hours exceeding US$95,000.^8^ Given the magnitude of these costs, we sought to conduct a formal economic analysis of fluvoxamine in the early treatment of COVID-19 to inform front-line COVID-19 mitigation strategies. Herein, we report a cost-consequence analysis based on the evidence provided by the largest of the published trials of fluvoxamine, the TOGETHER trial.^4^ We applied a decision-analytic model to evaluate the most cost-effective strategy for outpatient treatment of adult patients presenting with symptomatic COVID-19 and known risk factors for disease progression. Two treatment strategies were evaluated in the primary analysis: treatment with a 10-day course of fluvoxamine (100 mg twice daily) and current standard of care (SOC). A secondary analysis comparing a 5-day course of nirmatrelvir–ritonavir with SOC was also conducted.

## METHODS

### Decision model.

We applied findings from the TOGETHER trial^4^ to the U.S. hospital setting. Our modeled population included adults with confirmed COVID-19 infection who were at increased risk of progression to severe disease or hospitalization, based on established risk factors including age, obesity, and comorbidities. The patient cohort within the model transitioned through the care pathway over the course of 28 days, as this was the duration of follow-up for the trial’s composite primary endpoint. The model reported the level of healthcare utilization by cohort, including extended emergency department (ED) use, hospital admission, ICU admission, total length of hospital stay, and discharge. Long-term consequences of COVID-19 (i.e., with a clinical course exceeding 28 days) were not considered but warrant exploration and analysis when suitable clinical data are available.

We applied a U.S. healthcare system perspective in which third parties (insurers) reimburse for healthcare services through bundled payments; thus, only direct medical costs were considered (e.g., costs related to treatment acquisition, administration, and condition-related care). Productivity effects from the patient perspective and other indirect costs were not considered for this chosen perspective.

In light of the documented decrease in disease progression among patients who are given fluvoxamine and fluvoxamine’s safety and tolerability,^4^ we hypothesized that the results of a cost–utility analysis would show that fluvoxamine delivers greater health benefits at a lower total cost than SOC (i.e., that it dominates SOC). Because the value of the primary endpoint of preventing disease progression was well established, we did not consider quality-of-life measures and determined that a cost–consequence analysis was sufficient to address the study objective. Healthcare resource utilization, measured in terms of cost and length of stay, were considered the outcomes of primary relevance to resource planning decisions in the United States but were also considered valuable and interpretable for decision-making in other settings. Threshold analyses of the cost of hospital admission and ICU admission were conducted to identify the values at which the use of fluvoxamine became cost neutral.

**Figure 1.**
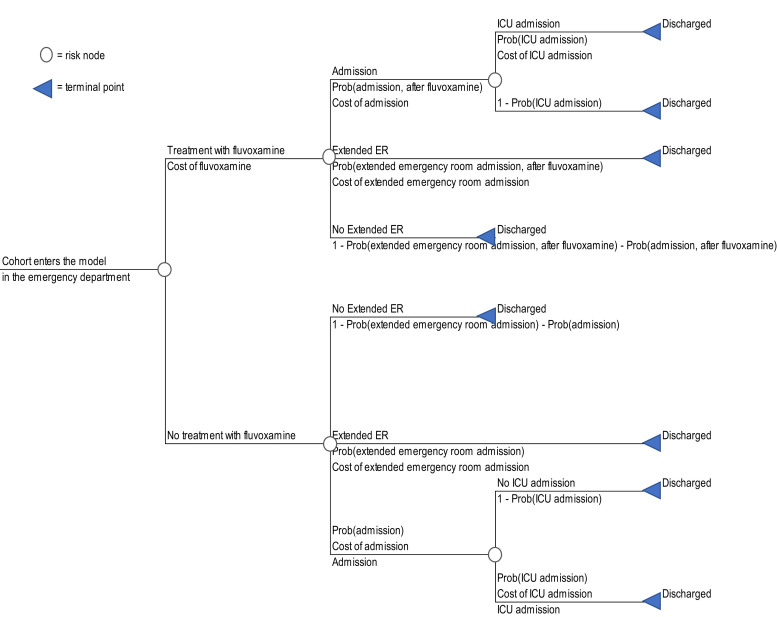
Model schematic.

Given the limited time horizon of the trial data and the fundamentally financial objectives of the analysis, we applied a decision-tree model. A simple arithmetic model was constructed in Microsoft Excel using the TreePlan add-in (TreePlan Software, San Francisco, CA). The 28-day time horizon obviated the need for discounting of costs or effects. A schematic of the model is shown in Figure 1.

### Input parameters.

Our decision-tree model allowed for the calculation of expected costs for each treatment strategy, programmed as mutually exclusive sequences of events or pathways through which the patient cohort passes.^9^^,^^10^ Expected values were calculated by summing the pathway values, weighted according to the conditional probability of each sequence of events.

Patient cohorts entered the model in the ED, with care escalating according to the rates reported in the intent-to-treat population of the trial publications, either in the form of extended ED stay or hospital admission. Because both cohorts entered the model in the outpatient setting, the costs of a routine ED visit were omitted because they would be the same for both cohorts. The cost of the extended ED visit was assumed to be 33% greater than the ED visit cost obtained from the Agency for Healthcare Research and Quality (US$608.46) because COVID-19 patients typically spend an average of > 3 hours in emergency settings.^11^ Given the uncertainty in this value, we also examined this parameter in a univariate sensitivity analysis. The total length of stay was calculated for each strategy and reported with the cost results. Once hospitalization has occurred, the use of fluvoxamine is not expected to affect the likelihood of ICU admission, and so the same risk was applied to both model arms. This approach is supported by a recent prospective open-label trial that reported no benefit from fluvoxamine on days on ventilator support, days in ICU, or total length of hospitalization.^12^ However, the approach differs from a recent analysis of molnupiravir, which used reduced rates of care escalation for treated patients based on post hoc analyses of the trial data.^13^

Clinical and economic data used in the model are reported in Table 1. All costs were inflated to 2021 U.S. dollars, using the Medical Care Inflation Calculator.^14^ The age distribution of hospitalizations for COVID-19 and the probability of ICU admission for each of those age groups were obtained from Bartsch and colleagues (2020).^15^ These values enabled the calculation of a weighted average for the probability of ICU admission, given hospitalization, and the weighted average cost for an emergency department visit. This value was then used to calculate the incremental cost associated with an extended emergency department visit.

**Table 1:** Clinical, economic, and resource inputs to model, all 2021 US dollars

	Default
Clinical inputs to the model	
Parameter	
Probability of extended ED stay, standard of care	0.0409
Probability of extended ED stay, fluvoxamine	0.0081
Probability of hospital admission, standard of care	0.1228
Probability of hospital admission, fluvoxamine	0.1001
Probability of admission to ICU, given if hospitalized	0.2224
Economic cost inputs to the model		
Drug costs		
Fluvoxamine 10-day course	$7.42
Nirmatrelvir–ritonavir therapy, 5 days	$529.00
Standard of care	$0.00
Hospital services costs		
ED visit	$608.46
Extended ED visit (incremental)	$200.79
Hospital admission	$6,956.08
ICU admission	$14,887.30
Length of stay for respiratory admission		
ED visit	0.0
Extended ED visit	0.0
Pneumonia with or without complications or comorbidities	3.1
RSD with < 96 hours of ventilator support	5.8
RSD with > 96 hours of ventilator support	22.6
RSD ICU admission	16.0

ED = emergency department; ICU = intensive care unit; RSD = respiratory system diagnosis.

**Figure 2.**
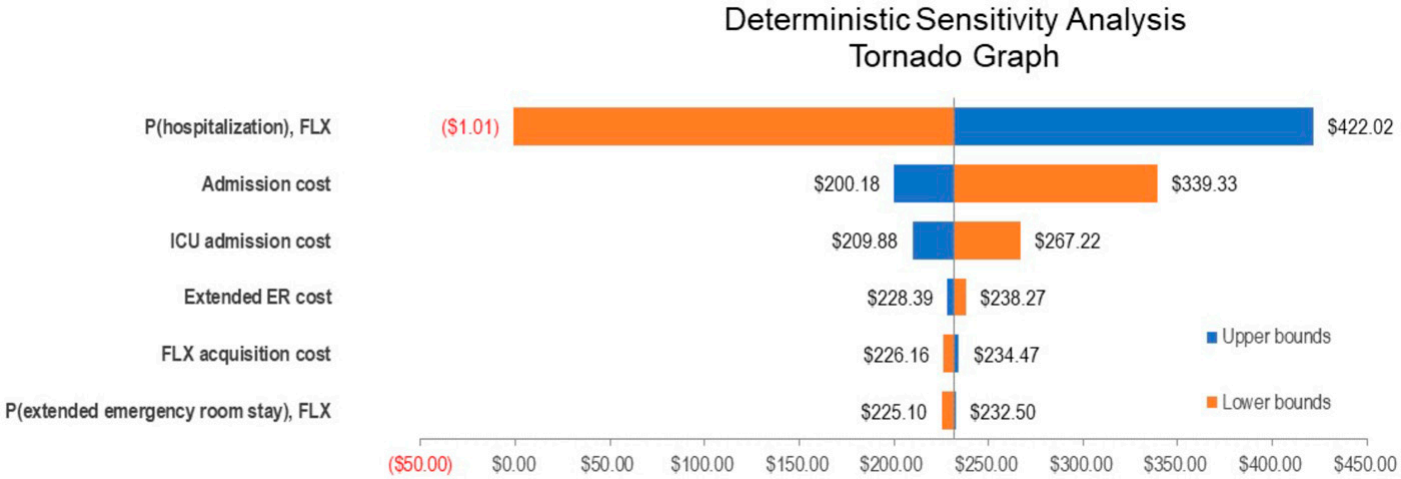
Tornado diagram for one-way sensitivity analyses.

Length of stay for each of the possible admission types were obtained from Rae et al (2020).^8^ Emergency visits, whether of normal duration or extended, were not assigned a hospitalization length of stay value because it is not clear whether this parameter affects hospital capacity. It has been estimated that 61% of patients admitted to ICU require ventilator support,^15^ for which we calculated a weighted average length of stay of 16 days. In addition to the primary assumptions reported in Table 1, one-way sensitivity analyses were conducted to assess the effect of individual parameter values and were presented in tabular form (Table 2) and as a tornado diagram (Figure 2). Inadequate data were available with which to inform a direct comparison of the efficacy of fluvoxamine and nirmatrelvir–ritonavir, owing to the different populations and event rates in their respective trials.^4^^,^^16^ As the competing direct-acting antivirals target earlier treatment of outpatients and are not yet provided in any published form, an indirect treatment comparison was not possible. However, in the interest of completeness, a secondary analysis has been conducted to inform the potential cost-savings of nirmatrelvir–ritonavir because it has been shown to be highly effective and has been approved by the U.S. Food and Drug Administration and other national and multinational regulators.^17^

**Table 2:** One-way sensitivity analysis, inputs, and results for fluvoxamine

Parameter	Base case values	Model inputs	
		Low	High
Probability (extended ED stay, fluvoxamine)	0.0081	0.0042	0.0409
Probability (hospitalization, fluvoxamine)	0.1001	0.08160	0.1228
Extended ED cost	$200.79	$100.40	$401.58
ICU admission cost	$14,887.30	$10,557.47	$21,943.28
Fluvoxamine acquisition cost	$7.42	$4.64	$12.95
Hospital admission cost	$6,956.08	$5,564.87	$11,708.97
Probability ICU (given hospital)	0.2224	0.1668	0.2780

ED = emergency department; ICU = intensive care unit.

A series of one-way sensitivity analyses were conducted on the base-case analysis, as shown in Table 2. The purpose of these analyses was to assess the effect of individual parameters on the results, using plausible alternate values for each. For the probability of an extended emergency visit with fluvoxamine, the lower bound was set at half the reported fluvoxamine rate from the TOGETHER trial, and the SOC rate was used for the upper bound. For the extended emergency visit cost, we used half and double the default value as the lower and upper bounds, respectively. For ICU admission cost, the lower bound used the reported value for *pneumonia admission with no complications or comorbidities*, whereas the upper bound used the same reported value for *admission with major complication* reported by Rae et al.^8^ The fluvoxamine acquisition cost was tested using the lowest and highest retail prices found for 100 units through online searches.^18^ The probability of ICU admission, given hospitalization, varied by ± 25%. Finally, the cost of hospital admission was varied from that of 80% of the default value to the value calculated for severe pneumonia, weighted according to the published age profile of hospital admissions (2020).^15^

Because nirmatrelvir–ritonavir has demonstrated considerable efficacy and is approved for use in multiple jurisdictions,^16^^,^^17^ we conducted a secondary analysis using the outcomes reported by Hammond and colleagues.^16^ The secondary analysis assumes the same effect (relative risk of 0.111) on extended ED and hospitalization. This was necessary because the event rates between Reis et al. and Hammond et al. suggest meaningful prognostic differences between the trial populations.^4^^,^^16^ As with the primary analysis, no effect on escalation of care (e.g., likelihood of ICU admission), is assumed once hospitalization has occurred. Pricing information was obtained from the Institute for Clinical and Economic Review.^7^

This analysis adheres to the *Consolidated Health Economic Evaluation Reporting Standards* (CHEERS) statement.^19^

## RESULTS

### Fluvoxamine decision analysis.

The primary results of our decision analysis are presented in Table 3, reflecting substantial cost-savings and reductions in hospital resource requirements associated with the use of fluvoxamine in the target population. The results suggest that the use of fluvoxamine reduces total days of hospitalization by 0.15 days per person, comprising 0.7 days of regular admission and 0.8 days of ICU care. Overall costs were reduced by $231.69 across the study population when compared with standard of care.

**Table 3:** Results of primary analysis

	Fluvoxamine	SOC	Savings
Cost category			
Emergency department	$1.63	$8.22	$6.59
Hospitalization	$696.55	$854.09	$157.54
Drug costs	$7.42	$0.00	($7.42)
ICU utilization	$331.53	$406.51	$74.98
Total costs	$1,037.13	$1,268.82	$231.69
Resource use			
Regular admission days	0.31	0.38	0.07
ICU days	0.36	0.44	0.08
Total hospital days	0.67	0.82	0.15

ICU = intensive care unit; SOC = standard of care.

### Fluvoxamine threshold analyses.

To inform resource allocation decisions in other settings and determine the values at which the use of fluvoxamine would be expected to be cost-neutral, we conducted two threshold analyses for the cost of hospital admission and the cost of ICU admission. The values were tested independently, and thus, there were no positive values for either parameter that would make fluvoxamine use cost-neutral. In fact, even with both values set to zero, fluvoxamine’s effect on ED costs still yielded cost savings. However, setting the costs for both extended ED visits and ICU admissions to zero yielded a threshold value of $328 for hospital admissions; setting the costs for both extended ED visits and hospital admissions to zero yielded a threshold value of $1475 for ICU admissions.

To account for the role of vaccination in reducing rates of hospitalization, we also estimated the relative effect of fluvoxamine when the overall likelihood of hospitalization was reduced. Holding other values constant, the baseline risk of hospitalization (0.123 for SOC and 0.100 for fluvoxamine), would have to be reduced by > 99% for fluvoxamine to become cost neutral.

### Nirmatrelvir–ritonavir scenario analysis.

The primary outcome of the nirmatrelvir–ritonavir trial^16^ differs from that of the TOGETHER trial because it is a composite of hospitalization or death and does not consider extended ED visits. At an anticipated price of $529 per 5-day course of treatment and assuming that the effect of nirmatrelvir–ritonavir on extended emergency visits is the same as its effect on hospitalization, our analysis suggests that nirmatrelvir–ritonavir is cost-saving in the amount of $625 per patient among those at high risk of progression to severe disease compared with SOC (Table 4), with a decrease of 0.74 hospitalization days.

**Table 4:** Scenario analysis: nitmatrelvir–ritonavir treatment, inputs, and results

	Nitmatrelvir–ritonavir	SOC	Savings*
Cost category			
Emergency department	$0.18	$8.22	–$8.04
Hospitalization	$77.32	$854	$776.77
Drug costs	$0.00	$529.00	–$710.00
ICU utilization	$406.51	–$369.71	$97.94
Total costs	$643.30	$1,268.82	–$625.52
Resource use			
Regular admission days	0.03	0.38	−0.35
ICU days	0.04	0.44	−0.40
Total hospital days	0.07	0.82	0.74

ICU = intensive care unit; SOC = standard of care.

* A negative value indicates cost-additive result.

## DISCUSSION

This study found that administration of a 10-day course of fluvoxamine to symptomatic COVID-19 outpatients at high risk of progression to severe illness is substantially cost-saving in the United States, saving US$232 and 0.15 hospitalization days per patient compared with SOC. Our secondary analysis found that nirmatrelvir–ritonavir was also substantially cost-saving, in the amount of $625, despite its higher acquisition cost. The regimen’s impressive efficacy, demonstrating an 89% reduction in hospitalization, more than compensates for its higher acquisition cost.

The potential cost-savings associated with fluvoxamine are unsurprising given the expected reduction in the incidence of hospitalization among those to whom it is administered, as well as its comparatively low cost, ease of administration, and innocuity. As illustrated in the tornado diagram (Figure 2), risk of hospitalization is the main driver of cost-savings because hospitalization is both an expensive and frequent occurrence among patients in the studies included in our model (reported in approximately 12% of studied patients in both the TOGETHER trial, and 10% in the more recent nirmaltrelvir–ritonavir trial). As noted in a meta-analysis by Lee et al.,^20^ the cost-savings associated with fluvoxamine are highly probable, yet other unproven drugs, such as ivermectin and hydroxychloroquine, continue to be prescribed. Although our analysis is based on data from the United States, these cost savings are likely to extend to other settings as well, including low- and middle-income countries, given the reductions in healthcare utilization. However, for health systems with lower nonpharmaceutical input costs, the savings from the combination regimen will be reduced, and price discounts would be needed to ensure its affordability.

Furthermore, the results of our analysis almost certainly understate the true value of fluvoxamine because ICU admissions generally incur substantial follow-up costs in the year after hospitalization,^15^ and these averted costs would not have been captured in our analysis. For instance, follow-up costs for acute respiratory distress syndrome, which is commonly diagnosed in ICU patients, amount to an estimated US$28,133 in the year post-hospitalization, and sepsis incurs US$10,531.^15^ Additionally, our model did not assign additional length of stay values for extended emergency visits, which may further underestimate the true resource savings and opportunities for efficiency presented by fluvoxamine use. Lastly, our analysis relied on the intent-to-treat analysis of the TOGETHER trial, rather than the per-protocol analysis (defined as the population of patients who took at least 80% of their prescribed drug or placebo). A per-protocol analysis yielded superior results and demonstrated a mortality benefit.

### Strengths and limitations.

Our study benefits from the fact that the results of a high-quality RCT were used to inform the analyses. However, as with all trials, the demographic and disease characteristics of the population enrolled may differ from those seen in other health systems, particularly in terms of vaccination status. As noted in a recent analysis of another COVID-19 treatment, molnupiravir,^13^ COVID-19 studies are inherently challenged by the evolution of the pandemic, particularly in relation to new viral strains and variability in SOC. However, subgroup analyses of the TOGETHER trial uniformly demonstrate favorable results for fluvoxamine, suggesting that heterogeneity of patient characteristics is unlikely to undermine the validity of the conclusions of this analysis, though further study of alternative SOC models is warranted.

Nonetheless, two sources of uncertainty affect the selection of appropriate patients for whom fluvoxamine would be effective. First, the TOGETHER study was conducted among predominantly unvaccinated patients, and further evidence is therefore needed to inform the clinical value and cost-savings of fluvoxamine among populations of different composition. Vaccinated patients over age 50 have a 2- to 3.5-fold lower risk of hospitalization compared with unvaccinated populations,^21^ which may impact the cost-savings associated with fluvoxamine administration. However, even with this reduction in risk of severe disease, fluvoxamine is highly likely to be cost saving, and a sensitivity analysis of the risk of treatment escalation suggested that the primary findings remain robust. Nonetheless, the evolution of the COVID-19 pandemic, involving both increased rates of vaccination and a shift toward newer viral strains, means that updates to this analysis with new clinical evidence will be required. Second, enrolled patients were not already receiving treatment with fluvoxamine, and it is unclear how many participants were receiving SSRIs more broadly. Given the widespread use of SSRIs in the United States, and the possibility that other drugs within the class may offer protection, further research is needed to determine whether patients already receiving such therapies should be expected to experience the same benefits as those observed in the TOGETHER study.

Although our model’s objective was to consider the impact of fluvoxamine on hospital resources, expressed as length of stay, a unit of measure easily interpreted by the intended audience, the inclusion of other utility-based measures such “equal-value life years gained,” or “quality-adjusted life years” would likely show fluvoxamine as dominant as well (i.e., producing greater population health benefits at a lower overall cost). Furthermore, the current model does not permit the consideration of a wider effect on the health system’s capacity or healthcare personnel, but it is likely that the avoidance of disease progression and attendant resource consumption, and indeed the increased mortality risk correlated with the level of respiratory support provided,^22^ would have substantial benefits in these areas, and this assessment is clearly needed.

In conclusion, by reducing the total costs and the need for escalation of care, the use of fluvoxamine among symptomatic COVID-19 outpatients at high risk of disease progression is substantially cost-saving. Given fluvoxamine’s tolerability, ease of use, affordability, and easy access, this finding has the potential to positively influence health system responses in the clinical management of COVID-19. Whether fluvoxamine is incorporated into the standard of care will depend on the setting in which it is being administered and the results of future clinical trials.
